# Environmental fate, biodegradation pathways, and phytotoxicological risks of biopolymer-based packaging: a critical review

**DOI:** 10.1007/s11356-026-37565-7

**Published:** 2026-03-06

**Authors:** Danielle Cristine Mota Ferreira, Camila Rodrigues Carneiro, José Roberto Miranda Júnior, Jane Sélia dos Reis Coimbra, Franciele Maria Pelissari, Eduardo Basílio de Oliveira

**Affiliations:** 1 Department of Food Technology, Universidade Federal de Viçosa, Viçosa, 36570-900 Brazil; 2 Institute of Science and Technology, Universidade Federal Dos Vales Do Jequitinhonha E Mucuri, Diamantina, 39100-000 Brazil

**Keywords:** Environmental safety assessment, Ecotoxicity testing, Compostability standards, Biopolymer end-of-life, Sustainable materials, Risk assessment

## Abstract

Biopolymer-based packaging has been widely promoted as a sustainable alternative to conventional plastics. However, biodegradability claims are often supported by incomplete assessments that prioritize disintegration or weight loss over actual mineralization, neglecting the ecotoxicological impacts of handling intermediates and waste. This review provides a critical and integrative analysis of current methodologies used to assess the biodegradation and environmental safety of biopolymer-based materials in different environments. The ecotoxicological discussion focuses on phytotoxicity as a sensitive indicator of environmental safety, while microbial processes are considered complementarily due to their central role in biodegradation. The review further discusses how environmental variations and polymer composition influence handling kinetics and toxicity results, highlighting the need for harmonized protocols that reflect real-world environmental conditions. Future perspectives highlight the integration of laboratory and field research, the application of advanced analytical and predictive tools (omics, sensors, artificial intelligence), and the proposal of a conceptual framework for “effective environmental degradability,” coupling mineralization parameters with ecotoxicological metrics. Overall, this work presents a comprehensive framework that connects biodegradation tests, phytotoxicity assessment, and regulatory interpretation, contributing to a more realistic understanding of the environmental fate and sustainability of biopolymer-based packaging.

**Graphical abstract:**

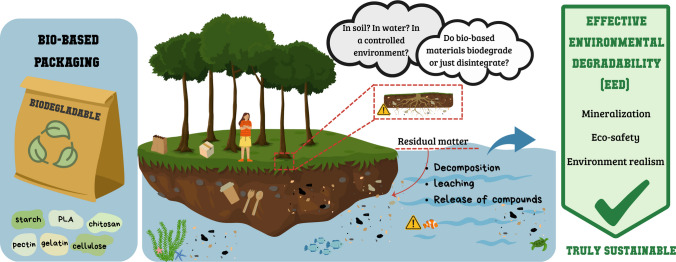

## Introduction

The accelerated production and inadequate disposal of conventional plastic waste represent one of the most pressing global environmental challenges, stimulating the search for alternatives aligned with circular economy principles. Among them, packaging derived from biopolymers, commonly referred to as bio-based packaging, has gained increasing attention as a promising solution to mitigate plastic pollution and reduce dependence on fossil resources (Barone et al. 2025). These materials can originate from renewable feedstocks such as corn, sugarcane, vegetable oils, wood, or algae, and in some cases even from agro-industrial by-products, which strengthens their appeal as contributors to a bioeconomy (Pelissari et al. 2018; Ferreira et al. 2020a, b, 2023).

However, the transition from petroleum-based plastics to biopolymer-based packaging is far from straightforward. The term bioplastic itself is ambiguous, often encompassing materials that are bio-based (derived from renewable sources), biodegradable, or both, while certain fossil-derived plastics are also marketed as “biodegradable,” creating conceptual and regulatory confusion (Bauer et al. 2024). The critical distinction lies between biodegradability, defined as the complete microbial mineralization of polymers into carbon dioxide, water, and biomass (Ammala et al. 2011), and mere disintegration, understood as fragmentation into smaller particles without microbial assimilation. The latter may generate biodegradable microplastics (BMPs) that persist in soil or aquatic ecosystems with poorly understood ecotoxicological consequences (Fan et al. 2025).

Current sustainability assessments of bioplastics, however, continue to privilege degradation kinetics as the main indicator of environmental safety. Standardized tests developed by ASTM, ISO, EN, and AFNOR organizations indeed allow reproducibility and regulatory control across composting, soil, aquatic, and landfill environments. Yet, these protocols focus almost exclusively on the disappearance of material mass or carbon conversion into CO₂/CH₄, rarely considering the ecotoxicological effects of degradation intermediates or additives released into the environment (Lin et al. 2025). This narrow approach risks equating “fragmentation” with “sustainability,” an assumption that is increasingly challenged by evidence of phytotoxicity and toxicity to soil microbiota arising from biodegradable polymers and their by-products (Zimmermann et al. 2020; Liwarska-Bizukojc 2022).

Therefore, a critical reassessment of how the environmental fate of bio-based packaging is evaluated is urgently needed. This review aims to: (i) clarify the conceptual distinction between biodegradation and disintegration; (ii) examine the strengths and weaknesses of the main standardized methodologies across different environments; (iii) highlight the neglected dimension of phytotoxicity as a determinant of environmental safety; and (iv) propose directions for integrating ecotoxicological endpoints and advanced analytical tools into new classification frameworks. By doing so, we seek to move beyond the simplistic measure of “time to disappearance” and toward a more comprehensive concept of effective environmental degradability, in which both degradation kinetics and ecological safety are equally considered.

## Methodologies for assessing biodegradability

### Biodegradability and disintegration: concepts and criteria

The development of biodegradable materials as a strategy to mitigate the environmental impacts associated with the accumulation of solid waste, particularly plastics, requires a precise understanding of the concepts of biodegradability and disintegration (Cheng et al. 2025; Hua et al. 2025). Although these terms are often used interchangeably, they describe distinct yet complementary phenomena, whose differentiation is essential both for the development of new materials and for their evaluation in accordance with international technical standards.

**Fig. 1:**
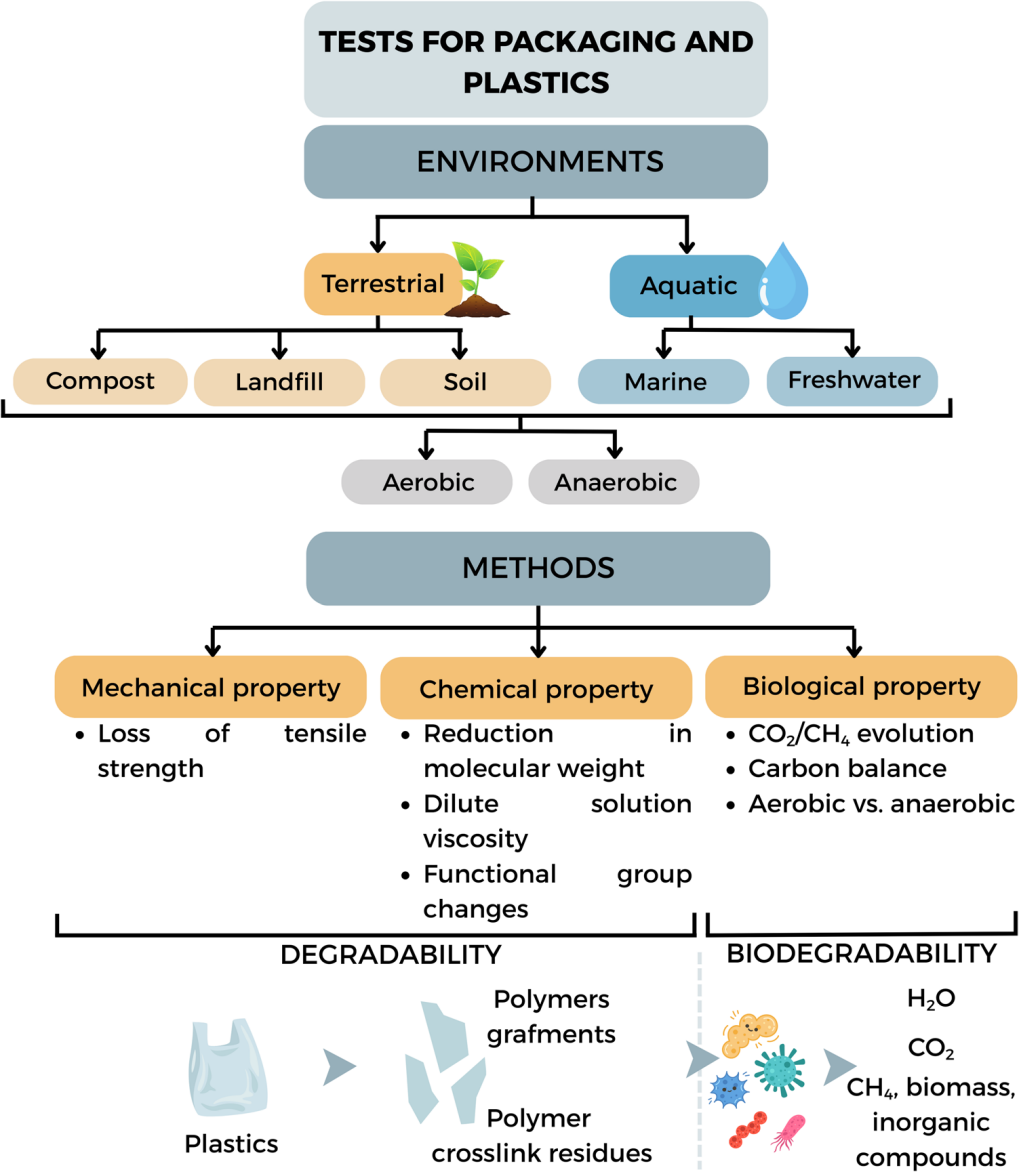
Classification of biodegradability and degradability tests for packaging and plastics across different environments and mechanisms

As illustrated in Fig. 1, biodegradability refers to the ability of a material to be biologically converted by microorganisms, including bacteria, fungi, and algae, into final products such as carbon dioxide (CO₂), water, biomass, and, under anaerobic conditions, methane (CH₄) (Kaur et al. 2025).

This process involves the depolymerization of macromolecular chains into oligomers or monomers, followed by microbial assimilation and metabolic conversion. Biodegradability depends not only on the material’s chemical composition but also on the environmental conditions under which degradation occurs, including temperature, moisture, oxygen availability, and the structure of the microbial community (Lucas et al. 2008; Hernández-García et al. 2021).

In contrast, disintegration refers to the physical fragmentation of the material into sufficiently small particles, driven by the combined action of physical, chemical, and biological processes. While disintegration does not necessarily imply the complete biological conversion of the material, it significantly increases the surface area, thereby facilitating subsequent microbial degradation (Laycock et al. 2017; Ortega et al. 2022). From a regulatory perspective, disintegration is typically assessed based on criteria such as the absence of visible residues or the retention of material fragments on standardized sieves following composting tests (ISO 20200; EN 13432; Afshar et al. 2025; Sarasa et al. 2009).

The distinction between biodegradability and disintegration is critical. A material may exhibit a high degree of physical disintegration without undergoing complete biodegradation, a phenomenon frequently observed in oxo-degradable plastics or polymers that fragment but produce chemically persistent residues (Yang et al. 2022; Chinglenthoiba et al. 2025). Therefore, the comprehensive evaluation of potentially biodegradable materials requires standardized, sensitive, and reproducible methodologies capable of quantifying not only physical fragmentation but also the effective conversion of organic carbon into mineralization end products (Laycock et al. 2017; Chinglenthoiba et al. 2025).

### Standards for biodegradation and disintegration of polymeric packaging materials

**Fig. 2:**
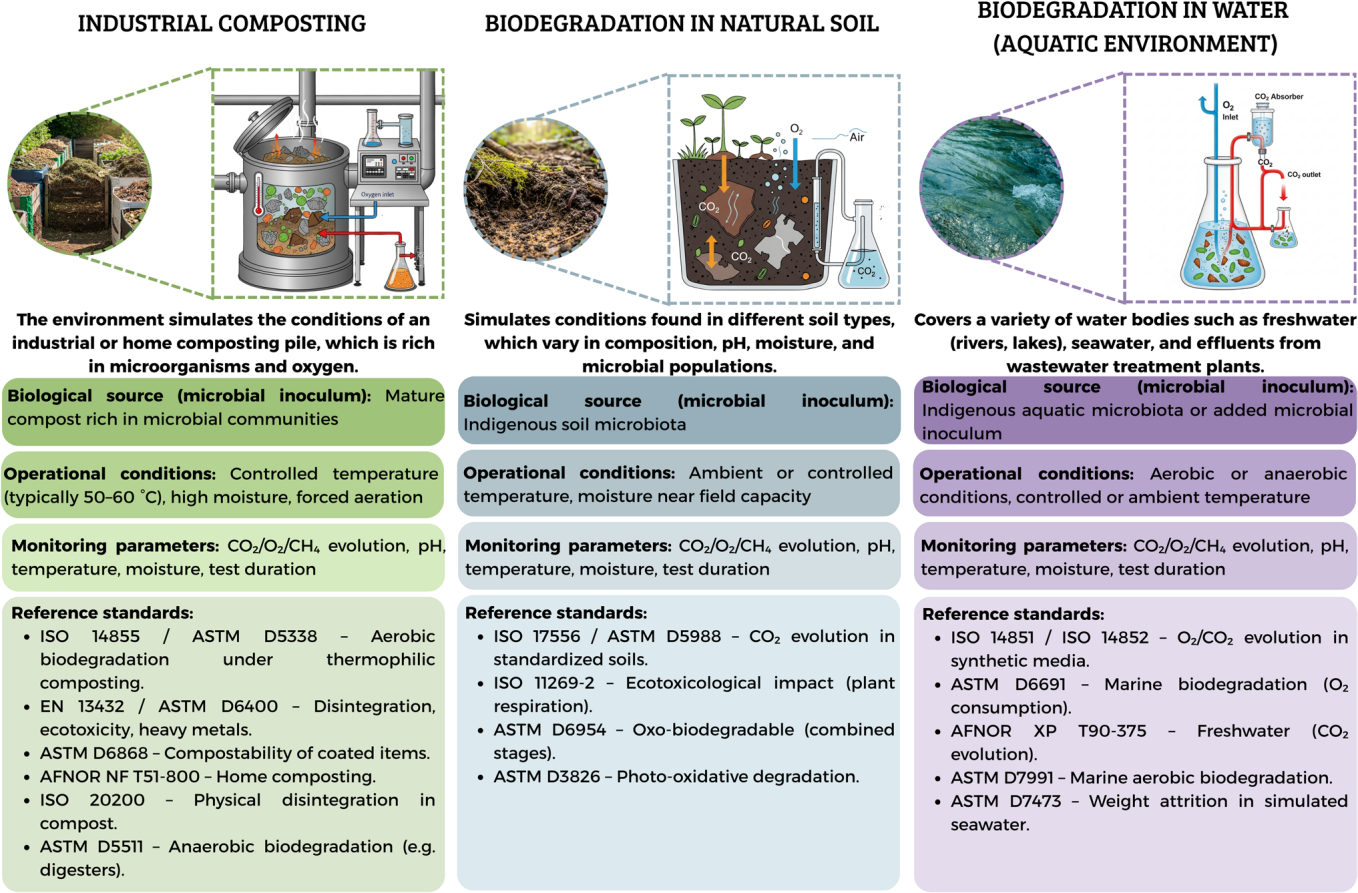
Classification of biodegradation test methodologies based on environmental simulation: controlled composting systems, standardized soil environments, and aquatic ecosystems

In general, biodegradation methodologies can be grouped into categories according to the simulated environment and degradation mechanisms: industrial composting, natural soil, aquatic systems (freshwater or marine), and landfill (anaerobic conditions), as illustrated in Fig. 2. This categorization is crucial to interpret not only the degradation performance of materials under distinct environmental conditions but also the ecological and regulatory relevance of each standard.

Given the structural diversity of polymeric materials and the heterogeneity of disposal environments, the development of standardized methodologies has become essential to ensure reproducibility and comparability. International organizations such as ISO, ASTM, CEN, AFNOR, and OECD play a pivotal role in providing technical guidelines applicable across composting, soil, and aquatic systems, and at different stages of packaging life cycles. These include ISO 14851 (1999), ISO 14852 (2018), AFNOR XP T90-375 (2012), ASTM D6691 (2017), ISO 17556 (2019), ISO 20200 (2015), ISO 11269–2 (2012), ISO 14855–1 (2012), ASTM D5338 (2015), EN 13432 (2000), ASTM D6400 (2021), ASTM D5988 (2018), ASTM D6868 (2019), AFNOR NF T51-800 (2015), ASTM D5511 (2018), ASTM D5526 (2018), ASTM D7475-11 (2020), ASTM D5526 (2018), ASTM D6954 (2024), ASTM D3826-98 (1998), OECD 301 (1992a, b, c, d, e, f) (A–F), OECD 302 (1981a, b, c) (A–C), OECD 306 (1992), OECD 310 (2014) which together encompass aerobic, anaerobic, and composting degradation scenarios. Table 1 summarizes these international standards, outlining their technical principles, advantages, and limitations.

**Table 1:** Standardized methods for assessing biodegradation and disintegration of polymeric materials in different environments

Standard	Application	Testing principle	Advantages	Limitations
*ISO 14851*	Aerobic biodegradation in static aqueous medium	Measurement of O₂ consumption or CO₂ production by microorganisms	Simple method; suitable for soluble or dispersible materials	May underestimate degradation of insoluble materials; low system homogeneity
*ISO 14852*	Aerobic biodegradation in aqueous medium with agitation	Similar to ISO 14851 but with continuous agitation	Improved contact between material and microorganisms; suitable for poorly soluble materials	Higher experimental complexity and operational costs
*AFNOR XP T90-375*	Freshwater biodegradability	Measurement of CO₂ evolution by microbial activity in freshwater environments	Directly applicable to freshwater litter scenarios; evaluates realistic environmental impact	Freshwater microbial communities are variable; sensitive to environmental fluctuations
*ASTM D6691*	Marine environments (seawater)	Measurement of O₂ consumption by native marine microorganisms	High relevance for materials disposed of in marine environments	Difficult standardization; highly variable and sensitive marine microbiota
*ISO 17556*	Aerobic biodegradation in natural soil	Measurement of CO₂ production by indigenous soil microorganisms	Representative of real environmental conditions; applicable to various polymers	High variability among soil types; influenced by external factors (moisture, temperature, microbial diversity)
*ASTM D5988*	Aerobic biodegradation in standardized soil	Monitoring CO₂ production during material incubation in soil	Standardized method; good reproducibility under controlled laboratory conditions	Results may not reflect degradation in natural soils; sensitive to soil composition
*ISO 11269–2*	Soil biodegradation (incubation test)	Measurement of microbial respiration (CO₂ evolution) in soil	Realistic assessment of soil degradation processes	Soil variability can affect results
*ISO 14855*	Industrial composting (controlled conditions)	Quantification of CO₂ released compared to a reference material under composting conditions	High reproducibility; widely accepted by certification bodies	Does not assess physical disintegration; not applicable to natural environments
*ASTM D5338*	Industrial aerobic composting (thermophilic, 58 °C)	Measurement of CO₂ evolution in reactors containing active compost	High accuracy; frequently used for compostability certification	Requires specific equipment; does not assess disintegration
*EN 13432*	Compostable packaging	Requires ≥ 90% biodegradation, physical disintegration, absence of toxicity, and limits on heavy metals	Comprehensive criteria for “compostable” labeling; internationally recognized by certifying bodies	Applicable only to packaging (not raw materials or pellets); multiple tests increase time and cost
*ASTM D6400*	Industrial composting (compostability requirements)	Requires ≥ 90% biodegradation within 180 days, disintegration within 84 days, and absence of toxicity	Integrates biodegradation, disintegration, and ecotoxicity; robust regulatory framework	Requires multiple tests; restricted to controlled industrial composting conditions
*ISO 20200*	Physical disintegration in simulated composting	Visual assessment of fragmentation and quantification of mass loss at 58 °C under aerated conditions	Evaluates physical disintegration; relatively rapid method	Does not measure chemical biodegradation; dependent on compost homogeneity
*ASTM D6868*	Compostable packaging with biodegradable coatings	Requires biodegradation of substrate and coating, physical disintegration, and absence of toxicity	Addresses multilayer materials; integrates chemical and physical degradation assessments	Applies primarily to coated packaging; requires comprehensive multi-step testing
*AFNOR NF* *T51-800*	Home composting certification	Requires ≥ 90% biodegradation, physical disintegration, absence of toxicity under home compost conditions	Relevant for household composting; more realistic for consumer use	Composting at home is less controlled; longer degradation times required
*ASTM D5511*	Anaerobic biodegradation (landfill simulation)	Measurement of methane and CO₂ production under anaerobic conditions	Representative of typical landfill conditions; applicable to oxo-degradable plastics	Requires strict control and specialized equipment
*ASTM D5526*	Anaerobic biodegradation under landfill conditions (long-term)	Measurement of methane and CO₂ over extended periods (6 + months) in anaerobic conditions	Representative of long-term landfill behavior; relevant for durable plastics	Long duration; requires specific anaerobic reactors and gas monitoring; high operational cost. |
*ASTM D7475*	Landfill simulation (sequential aerobic–anaerobic)	Assessment of aerobic oxidative degradation (mechanical property loss) and anaerobic biodegradation (CH₄ and CO₂ production)	Simulates landfill aerobic-to-anaerobic transition; evaluates both degradation phases	Complex setup; requires sequential aerobic and anaerobic stages
*ASTM D6954*	Guide for oxo-biodegradable plastics	Combined evaluation of abiotic degradation, biodegradation, and ecotoxicity	Holistic approach; applicable under various conditions	Functions as a guideline (not a specific method); requires complementary standardized tests for validation
*ASTM D3826*	Photo-oxidative degradation of plastics	Assessment of physical property loss after exposure to heat and UV radiation	Suitable for screening photo-oxidative degradation behavior	Does not measure biodegradation; only abiotic degradation pathways
*OECD 301 (A–F)*	Ready biodegradability screening under aerobic conditions	Carbon dioxide evolution, biochemical oxygen demand, or dissolved organic carbon removal, “10-day window” concept	Widely used in screening/regulation; clear criteria	Harsh conditions can generate false negatives for materials that degrade, but not “quickly.”
*OECD 302 (A–C)*	Intrinsic Biodegradability	Less severe conditions (higher inoculum/time), assesses intrinsic potential	Reduces false negatives compared to conventional biodegradability tests	Does not equate to rapid biodegradation in the environment
*OECD 306*	Marine biodegradation	Aerobic biodegradation in seawater (endpoint similar to CO₂/BOD, depending on the arrangement)	Relevance to marine litter	High variability of inoculum/matrix
*OECD 310*	CO₂ headspace test	Measurement of CO₂ in headspace under standardized conditions	Direct mineralization endpoint (CO₂)	Still a screening test; does not replace “realistic” scenarios

The evaluation of industrial composting is more consolidated from a methodological perspective. Standards such as ISO 14855 and ASTM D5338 simulate thermophilic composting conditions (58 °C) under strict control of moisture and aeration, using active compost (typically derived from municipal organic waste or green waste rich in mature microbial communities) as the microbial substrate. These tests offer high sensitivity and reproducibility and are widely accepted for regulatory purposes. However, they do not represent natural environments and should be interpreted considering the local waste management infrastructure (ASTM D5338-15; ISO 14855-1:^2012^; Jung et al. 2025).

More comprehensive standards such as EN 13432 and ASTM D6400 establish integrated criteria for biodegradation, disintegration, ecotoxicity, and heavy metals, providing broad certification frameworks for compostable packaging (ASTM D6400-21; ASTM D6868-19; EN 13432:^2000^; Shruti et al. 2020; Samneingjam et al. 2025). Complementing these, ASTM D6868 addresses the increasing use of multilayer materials, requiring both substrate and biodegradable coatings to comply with compostability standards under industrial composting conditions (ASTM D6868-19; Bher et al. 2023).

Additionally, AFNOR NF T51-800 establishes criteria for home composting, which operates under milder, less controlled conditions, demanding longer degradation times (AFNOR NF T51-800:^2015^; Nhu et al. 2024). Together, these standards are essential to ensuring the safety and efficiency of the organic recycling process. However, the multiplicity of required tests increases the time and cost of certification, posing barriers for small producers or materials in early stages of technological development (Afshar et al. 2025).

Standards such as ISO 20200, focused on evaluating physical disintegration, provide important complementary information, particularly regarding the fragmentation of materials under simulated composting conditions (ISO 20200:^2015^; Afshar et al. 2025). Although these tests do not directly assess the microbial conversion of polymeric carbon, they are crucial to ensuring that materials do not leave visible residues in the final compost.

In addition to ISO, ASTM, AFNOR, and CEN standards, the Organisation for Economic Co-operation and Development (OECD) provides a widely adopted guideline framework that supports biodegradability and persistence assessments in regulatory contexts. OECD guidelines are commonly organized into a tiered strategy comprising ready biodegradability tests, inherent biodegradability tests, and simulation tests, reflecting increasing environmental realism and analytical complexity. In the ready tier, biodegradation is typically assessed using carbon dioxide (CO_2_) evolution, biochemical oxygen demand (BOD), and dissolved organic carbon (DOC) removal, with predefined pass criteria and interpretation concepts such as the “10-day window” after biodegradation onset. This framework is closely connected to ISO-based approaches and helps standardize interpretation across studies, while also clarifying limitations of screening assays, including the lower specificity of DOC removal compared with CO_2_ or BOD and the potential for false-negative outcomes under stringent test conditions.

Building on these compartment-specific standards, for soil environments, ISO 17556 and ASTM D5988 adopt similar strategies, measuring CO₂ evolution as an indicator of microbial activity on the material mixed with soil (ASTM D5988-18; ISO 17556:^2019^). The primary advantage of these methods lies in their attempt to simulate uncontrolled disposal in terrestrial environments, which is particularly relevant in regions with limited waste management infrastructure. Nonetheless, the inherent variability of soils, including pH, organic matter content, moisture, mineral composition, and microbial diversity, compromises test reproducibility and complicates the extrapolation of results (Lakshmi and Saravanakumar 2024).

ISO 11269-2, although originally designed for assessing toxicity in agricultural soils, has also been applied to investigate the effects of biodegradable materials on microbial respiration. It thus complements biodegradation tests with a more ecological focus, allowing the evaluation of potential impacts on soil health following polymer fragmentation or mineralization (ISO 11269-2:^2012^; Yu et al. 2024).

In aquatic environments, standards ISO 14851 and ISO 14852 employ the quantification of oxygen consumption or CO₂ evolution as indirect indicators of aerobic biodegradation (ISO 14851:^1999^; ISO 14852:^2018^; García-Depraect et al. 2022; Poli et al. 2025). ISO 14852 incorporates continuous agitation, enhancing contact between the test material and the microbial community, making it more suitable for poorly soluble polymers (García-Depraect et al. 2022). However, both operate under controlled laboratory conditions, which limits their environmental representativeness, especially when compared to natural aquatic ecosystems where factors such as UV radiation, salinity, hydrodynamic flow, and biofilm formation significantly influence degradation processes.

The AFNOR XP T90-375 and ASTM D6691 standards represent a significant improvement by employing natural freshwater and seawater, respectively, with native microbiota (AFNOR XP T90-375:^2012^; ASTM D6691-17). While AFNOR assesses CO₂ evolution in freshwater, ASTM evaluates O₂ consumption under marine conditions. Both offer more realistic scenarios than synthetic media but face limitations in standardization due to microbial and chemical variability in natural waters (Schmid et al. 2021).

ASTM D5511 and ASTM D7475 extend biodegradability assessments to anaerobic environments, particularly landfills (ASTM D5511-18; ASTM D7475-11, 2020). ASTM D5511 quantifies CH₄ and CO₂ production under controlled anaerobic conditions, providing critical data on the chemical biodegradation potential of materials in final disposal scenarios (Samneingjam et al. 2025). This method demands rigorous control of operational parameters, including pH, moisture, temperature (35–37 °C), and organic loading, as well as specialized equipment such as gas-tight anaerobic reactors with integrated gas collection systems.

In parallel, ASTM D7475 evaluates the sequential behavior of materials under simulated landfill bioreactor conditions, encompassing an initial aerobic degradation phase, characterized by oxidative chain scission and loss of mechanical integrity, followed by anaerobic biodegradation under methanogenic conditions. This two-step approach provides comprehensive insights into both the abiotic oxidative degradation and the subsequent biological anaerobic mineralization of plastics (ASTM D7475-11; Rashidi 2022).

Standards ASTM D6954 and ASTM D3826 address key aspects of oxo-biodegradable plastics (ASTM D3826-98; ASTM D6954-24). ASTM D6954 provides guidelines for evaluating these materials by combining abiotic degradation (heat, light, oxygen), aerobic biodegradation, and ecotoxicity assessment of residues. As a guideline rather than a standardized test, it requires complementary methods for comprehensive validation (Moreno et al. 2023).

ASTM D3826 evaluates photo-oxidative degradation by measuring the loss of mechanical properties such as tensile strength, elongation at break, modulus of elasticity, and impact resistance in plastics exposed to heat and UV radiation (ASTM D3826-98; Ebrahimi et al. 2019). Although it does not assess biodegradation directly, this standard is crucial for materials designed to undergo abiotic degradation before microbial assimilation.

Recent European policy frameworks have accelerated the demand for harmonized standards and more transparent environmental assessments of packaging materials. The European Green Deal (COM/2019/640) and the EU Packaging and Packaging Waste Regulation (PPWR, COM/2022/677) explicitly promote a shift toward reusable and truly recyclable or biodegradable packaging, linking product claims to measurable environmental performance. These initiatives have intensified the revision of existing compostability and biodegradability standards under CEN/TC 261 and ISO/TC 122, driving the integration of ecotoxicity testing and real-world validation into regulatory procedures. As a result, methodological convergence is no longer only a scientific necessity but also a regulatory imperative to ensure consistency, transparency, and credibility across national certification schemes (CEN/TC 261 (2023).; European Commission (2019); European Commission (2022).; ISO/TC 122. (2023)).

Therefore, it is clear that the appropriate selection of standards for biodegradation and disintegration testing depends not only on the intrinsic properties of the materials but also on their expected environmental fate and the regulatory requirements of the target market. Standards based on industrial composting are more applicable within urban and regulatory contexts, whereas those targeting soil and aquatic environments are more representative of uncontrolled environmental dispersion scenarios. Whenever feasible, the combined application of different methodologies provides a more robust overview of the environmental behavior of materials, contributing to the development of truly sustainable packaging solutions.

## The emergence of phytotoxicity assessment

In the context of biodegradable packaging, phytotoxicity refers to the potential of degradation by-products to impair seed germination, root elongation, biomass accumulation, and physiological processes such as photosynthesis (Liwarska-Bizukojc 2022).

If on the one hand this review emphasizes phytotoxicity parameters, on the other hand it is important to recognize that microbial toxicity is a complementary component of environmental safety and often mechanistically upstream of biodegradation outcomes, because microorganisms drive biodegradation and support key functions in soil and wastewater systems. As recently reviewed by Strotmann et al. (2024), standardized approaches include activated sludge respiration inhibition tests (for general microbial activity) and nitrification inhibition tests (for sensitivity of the nitrogen cycle), which are widely used in regulatory contexts to detect inhibition that may confound biodegradation results or reveal the toxicity of degradation products. Furthermore, rapid assays with luminescent bacteria (often referred to as Microtox-type tests) allow sensitive screening of aqueous extracts, leachates, or process effluents where complex mixtures and transformation products are expected. From the perspective of end-of-life management of bio-based packaging, these microbial parameters are particularly relevant for evaluating compost extracts, soil leachates, landfill leachates, and wastewater treatment interfaces, where inhibitory effects can arise even when macroscopic disintegration is observed.

To further align this discussion with internationally harmonized regulatory tools, OECD test guidelines provide well-established reference methods for both microbial and plant-related ecotoxicity endpoints. For microbial communities, OECD 209 (2010) (Activated Sludge, Respiration Inhibition Test) is widely used to detect inhibition of overall heterotrophic activity, while OECD 216 (2000) (Nitrogen Transformation Test) and OECD 217 (2000) (Carbon Transformation Test) address soil microbial functioning directly linked to nutrient cycling and ecosystem services. In parallel, plant-focused OECD frameworks such as OECD 208 (2006) (Terrestrial Plant Test: Seedling Emergence and Seedling Growth) and OECD 227 (2006) (Terrestrial Plant Test: Vegetative Vigour) provide standardized endpoints that strengthen the interpretation of phytotoxicity data in soil–plant systems. Integrating OECD-based microbial and plant assays is particularly valuable when assessing biodegradable packaging residues, extracts, or leachates, because inhibition of microbial processes can directly affect mineralization and nutrient cycling, mediate downstream effects on plant performance, and also confound biodegradation outcomes.

Nevertheless, the ecotoxicological picture remains incomplete if plant-level endpoints are overlooked. Although well established in studies with conventional plastics and microplastics, phytotoxicity remains largely neglected in the assessment of bio-based packaging. Most end-of-life research still focuses on biodegradation or disintegration rates as primary indicators of environmental compatibility, rarely including biological assays that measure the effects of degradation products. As a result, a material may fragment or even mineralize while still releasing soluble compounds, additives, or intermediates capable of harming soil health and plant development. Existing literature is therefore dominated by studies on generic microplastics, with few evaluations targeting biopolymer-based packaging. Bridging this gap is crucial to ensure that the assessment of “biodegradable” packaging extends beyond mass disappearance to genuine ecological safety. Moreover, when inadequately discarded, biopolymers may undergo incomplete biodegradation and remain exposed to physical, chemical, and biological processes that promote progressive fragmentation, ultimately generating biodegradable microplastics (BMPs), particles derived from nominally biodegradable materials that fail to achieve full mineralization (Vargas-Estrada et al. 2025; Fan et al. 2025; Moudache et al. 2025).

BMPs contribute to the persistence of bioplastic pollution and can negatively impact terrestrial and aquatic ecosystems, which act as important sinks for these particles (Shi et al. 2024). These effects have raised growing concerns about the sustainability and ecological integrity of global ecosystems (Xu et al. 2020; Li et al. 2023).

The classification of bioplastics as biodegradable, environmentally safe, or ecotoxicity-free cannot be generalized, regardless of the environment, concentration, or chemical composition, without specific tests to assess their potential ecotoxicological risks (Goel et al. 2021). In this context, plants play an essential role in the formation and stability of soil structure, the dynamics of microbial communities, and the maintenance of ecosystem functions.

Some plant species are particularly sensitive to contaminants, and the effects of exposure tend to be most pronounced in the early stages of development, such as germination and seedling growth (Dissanayake et al. 2022). Several studies have reported the impacts of microplastics on key processes, including germination, biomass accumulation, root growth, and shoot elongation (Li et al. 2023; Chen et al. 2025). Furthermore, the interaction of BMPs with microorganisms and the biota of the soil–plant system can modify nutrient availability and, indirectly, compromise plant growth. Therefore, assessing the phytotoxicity of biopolymers applied to packaging emerges as a still underexplored but essential tool for detecting and monitoring potential ecotoxicological effects, functioning as a sensitive bioindicator of the presence and impact of contaminants in the environment.

Studies have shown that biodegradable microplastics (BMPs), including polyhydroxyalkanoates (PHA), polylactic acid (PLA), poly(butylene adipate-co-terephthalate) (PBAT), and polyhydroxybutyrate (PHB) derivatives can negatively impact the growth of several plant species under soil–plant exposure scenarios typically tested at 0.02–2.0% (w/w; dry soil basis), and in some cases up to 10% (w/w) to represent high-exposure conditions. Fan et al. (2025), for instance, evaluated BMP additions of 0.1% and 1.0% (w/w) and reported significant effects of PHA, PLA, and PBAT microplastics on biomass, shoot length, and chlorophyll-related parameters in tomato (*Solanum lycopersicum* L.). Also, Han et al. (2024) tested PBAT microplastics at 0.02%, 0.2%, and 2.0% (w/w) and observed significant reductions in pakchoi (*Brassica chinensis* L.) shoot and root biomass. In addition, Serrano-Ruiz et al. (2023) reported that biodegradable PHB-based mulch/coating materials severely inhibited tomato and lettuce (*Lactuca sativa* L.) growth, highlighting that phytotoxic outcomes may also arise from film-based exposure and weathering conditions, not only from soil-mixed particles.

Studies have also suggested that BMPs may be more harmful to plants than conventional microplastics under comparable loadings. For example, Qi et al. (2018) used 1% (w/w) of mulch-film residues (low-density polyethylene, LDPE, versus a starch-based biodegradable film) and observed impaired wheat (*Triticum* *aestivum* L.) growth with the biodegradable treatment, while LDPE showed no significant effects. Likewise, Sun et al. (2023) compared polyethylene (PE) and biodegradable microplastics at 0.1%, 1%, and 10% (w/w) and reported stronger toxic effects of biodegradable particles on maize (*Zea mays* L.) than PE under the same concentration range.

Table 2 presents the effects of BMPs on seed germination, plant growth, and rhizosphere microbiota in soil–plant systems, with an emphasis on agricultural species of broad importance in human nutrition.

**Table 2:** Effects of biodegradable microplastics (BMPs) on seed germination, plant growth, and soil/rhizosphere parameters in different plant species

Plant species	BMP type and concentration	Particle size (µm)	Exposure time	Tests performed	Main effects	References
Barley (*Hordeum vulgare*)	Starch-PBAT mixture 0.01, 0.1, and 1% in soil–plant system (pH 6.0 ± 0.1)	50–1000	4–14 days	Seed germination, plant growth, and chlorophyll content	No effect at 4 d; at 14 d: ↓ germination (25%) and biomass	(Zantis et al. 2024)
Wheat (*Triticum aestivum *L.)	Starch-PBAT mixture 0.01, 0.1, and 1% in soil–plant system (pH 6.0 ± 0.1)	50–1000	4 days	Germination, plant growth, and chlorophyll content	No effect on germination; ↓ root length (48%) and shoot weight	
Soybean (*Glycine max *(L.)* Merr*.)	PBAT 0.1%, 0.2%, 0.5%, and 1% in soil–plant system (pH 8.48)	250–5000	Not reported	Root growth and soil enzyme activities	Inhibited root biomass; altered soil enzyme activity	(Yu et al. 2023)
Sorghum (*Sorghum saccharatum* L.)	PLA and PHB 0.02; 0.095; 0.48; 2.38; 11.9% in soil–plant system (pH 6.0 ± 0.5)	2000–5000	3 days	Seed germination and plant growth	No effect on germination or growth	(Liwarska-Bizukojc 2022)
Rice (*Oryza sativa* L.)	PLA 10% in soil–plant system (pH 6.5)	155–180	30 days	Plant growth, exoenzyme kinetics, and microbial communities	↓ plant growth; ↑ soil enzyme activities	(Yu et al. 2023)
Common bean (*Phaseolus vulgaris* L.)	PBAT (85%) + PLA (10%) mixture at 0.5, 1, 1.5, 2.0, 2.5% in sandy soil (pH 6.0)	250–1000	105 days	Plant growth	↓ shoot biomass (≥ 1.5%); ↑ root traits and pod production at 2.5%	(Meng et al. 2021)
Carrot (*Daucus carota* L.)	PBAT 0.01, 0.1, and 1% in soil–plant system (pH 6.0 ± 0.1)	50–1000	10 days	Germination, plant growth, and chlorophyll content	No effect on germination; ↓ shoot length and biomass	(Zantis et al. 2024)
Tomato (*Solanum lycopersicum* L.)	PLA, PHA, and PBAT 0.1 and 1.0% in soil–plant system (pH 8.04–8.17)	100	60 days	Plant growth, soil properties, and rhizosphere microbiota	↓ shoot biomass; altered chlorophyll ratio and soil microbiota	(Fan et al. 2025)
Lettuce (*Lactuca sativa* L.)	PBAT 0.1 and 1.0% in moist soil–plant system (pH 8.47)	1.5–73	30 days	Plant growth, physiology, and soil properties	Dose-dependent ↓ shoot biomass, chlorophyll, and microbial activity	(Wang et al. 2024)
Pakchoi (*Brassica chinensis* L.)	PBAT 0.02, 0.2, 2% in soil–plant system	100–180	35 days	Plant growth, soil properties, and rhizosphere microbial communities	At 2%: ↓ shoot length (37%) and biomass (70%); altered rhizosphere microbiota	(Han et al. 2024)
Dandelion (*Taraxacum mongolicum Hand.-Mazz*.)	PBAT and PLA 1% in soil (pH 7.87)	< 5000	50 days	Plant growth, physiology, and metabolomics	↓ dry biomass (25–29%); altered metabolism and phenolic biosynthesis	(Li et al. 2024)

*BMPs* biodegradable microplastics, *PBAT* poly(butylene adipate-co-terephthalate), *PLA* polylactic acid, *PHA* polyhydroxyalkanoates, *PHB* polyhydroxybutyrate, *MPs* microplastics, *w/w* weight by weight, *pH* potential of hydrogen, no significant effect = no statistically significant effect compared to the control treatment (0% BMPs)

The effects are strongly modulated by plant species, concentration, and exposure time. Higher concentrations tend to significantly reduce the growth and biomass of several crops, such as common bean and pakchoi, while low concentrations show limited or no effects on species such as barley, wheat, and carrots during the early stages of development (Meng et al. 2021; Zantis et al. 2024).

Exposure time was also crucial, as short-term experiments (3–10 days) often showed no significant impacts, while prolonged exposures (≥ 30 days) resulted in reduced biomass, altered root and stem growth, and modifications to the rhizosphere microbiota, as observed in lettuce and tomato (Fan et al. 2025). Furthermore, the response varied between species, indicating physiological differences and differences in interaction with the soil microbiota. Sorghum maintained germination and growth even at high concentrations, while lettuce, beans, and pakchoi showed marked adverse effects (Liwarska-Bizukojc 2022).

In summary, data from previous studies reinforce that the impacts of BMPs on plants and microbiota in soil-plant systems are dose-dependent, species-specific, and cumulative over time, highlighting the importance of evaluating these variables when analyzing the environmental risk of biodegradable microplastics. This evidence has direct implications for sustainable agriculture, suggesting that the introduction of BMPs into soil should be carefully monitored, considering not only their biodegradability but also their potential adverse effects on agricultural crops and soil ecosystem health.

### Germination tests of seeds exposed to microplastics

Germination tests generally involve the following steps: (i) initial seed selection and preparation; (ii) preparation of microplastics and test solutions; (iii) seed exposure and germination; and (iv) data recording and calculation of germination rates.

In the initial step (i), good-quality seeds of known origin are selected and stored in dry conditions at a controlled temperature until use. Subsequently, the seeds are surface sterilized using 2–3% v/v hydrogen peroxide for 30 min (Sanna et al. 2022), sodium hypochlorite (Sun et al. 2023), or a combination of ethanol and sodium hypochlorite (Davoudpour et al. 2023), in all cases without significant negative effects on germination. After the exposure time, it is common practice to perform successive washes with sterile water (autoclaved distilled water or deionized water) to remove residues of the disinfectant agent, followed by drying the seeds under aseptic conditions, carried out in an oven at approximately 37 °C, under airflow, or on sterile paper/laminar flow in a controlled environment, in order to avoid excess moisture before sowing (Sanna et al. 2022).

In the microplastic and test solution preparation step (ii), commercial particles should be washed in water and/or methanol to remove loose additives, if necessary. If laboratory plastic fragments are used, their size is standardized by sieving or grinding and screening. The particles are then dried in a ventilated or vacuum oven (40–60 °C) (Lasota et al. 2024; Macan et al. 2024). Microplastic suspensions (MPs) should be prepared at the concentrations chosen according to the experimental design in sterile distilled water, ensuring homogeneity by vortexing or ultrasound (He et al. 2023).

Then (iii), each Petri dish is lined with sterile filter paper, to which a fixed volume of the MPs suspension or sterile water control is added (usually 5 mL in 9 cm diameter dishes), taking care to avoid waterlogging (Macan et al. 2024). A fixed number of seeds is placed on each plate, using at least four replicates per treatment to ensure statistical robustness (Lozano et al. 2022). The plates are then incubated at a temperature appropriate for the species under study (typically 20–25 °C for vegetables and 25–28 °C for tomatoes), with a photoperiod adjusted according to the physiological requirements of the plant, usually 12 h light/12 h dark or continuous dark (Jia et al. 2023). During the experimental period, the number of germinated seeds is recorded daily, considering radicle emergence ≥ 2 mm as the criterion (Ranal and Santana 2006).

Whenever necessary, small drops of sterile water are added to maintain the filter paper moisture (He et al. 2023). In the alternative procedure, MPs are mixed with dry soil in the desired proportion (% w/w) and homogenized (Sahasa et al. 2023). Then, pots or trays are filled with the treated substrate, sowing a fixed number of seeds per unit (usually 10–20), with at least four replicates per treatment. After sowing, irrigation with sterile water is performed, and the experimental units are incubated under controlled light and temperature conditions, with seedling emergence recorded daily (Lozano et al. 2022).

In the data recording and germination indices stage (iv), the most commonly used germination criterion considers a seed to have germinated when the radicle visibly emerges, usually with a length ≥ 2 mm, or another standardized value for the species (Ranal and Santana 2006). During the experiment, the day or time of germination of each seed is recorded daily, allowing the calculation of cumulative germination indices based on data from previous studies. Several indices have been proposed to translate germination patterns into comparable metrics, each with specific applications and limitations (Table 3).

**Table 3:** Germination indices commonly used in phytotoxicity assays

Index	Equation	Parameters	Description/purpose	References
Germination percentage (G%)	$\left(G\mathrm{\%}\right)=\left(\frac{{N}_{f}}{{N}_{t}}\right)x 100$	*Nf*: number of seeds germinated at the end of the test; *Nt*: total number of seeds sown	Proportion of germinated seeds relative to the total sown	(Ranal and Santana 2006)
Germination index (GI%)	$\left(GI\mathrm{\%}\right)=\sum \frac{{G}_{i}}{{T}_{i}}$	*Gi*: number of seeds germinated on day *i*; *Ti*: time (days) corresponding to day *i*	Measures germination weighted by time; often used in stress studies	(Farooq et al. 2005; Seo et al. 2025)
Mean germination time (MGT)	$MGT =\frac{\sum {n}_{i} x {t}_{i}}{\sum ni}$	*ni*: number of seeds germinated at time *ti*; *ti*: time (days) of germination	Weighted average germination time; lower values indicate faster germination	(Ellis 2022; Shi et al. 2024)
Vigor index (VI)	$Vigor\;Index=G\%\; x \;a\text{verage root}/\text{shoot length }(\mathrm{mm})$	*G%*: germination percentage; root/shoot length: mean seedling length in mm	Combines germination percentage with initial seedling growth	(Abdul‐Baki and Anderson 1973; Seo et al. 2025)

Other indices used include the Germination Velocity Index (GVI), which gives greater weight to early germination (Maguire 1962); the synchronization or uniformity of germination, which assesses the simultaneous germination of seeds within the same treatment (Finch-Savage and Bassel 2016); the relative germination rate index (GRI), which relates daily germination to cumulative time (Ranal and Santana 2006); and the T50, the time required for 50% of seeds to germinate (Farooq et al. 2005). These indices allow comparison not only of the proportion of germinated seeds, but also of the speed and uniformity of germination between treatments.

### Responses of the soil–plant system to the presence of microplastics: plant growth and soil microbiota

After germination, seedlings are transferred to controlled cultivation in pots or experimental soil, allowing growth and development to be monitored under standardized conditions. To evaluate the effects of microplastics on the soil–plant system (soil collected from the surface layer between 0 and 20 cm deep), they can be incorporated in two ways: directly mixed with dry soil, in proportions generally below 12% (w/w) and carefully homogenized (Liwarska-Bizukojc 2022), or as a suspension applied to previously moistened soil, ensuring contact with the root zone without waterlogging.

Pots or trays are filled with the treated substrate (soil-microplastics), and a fixed number of seedlings are transplanted per experimental unit, with at least four replicates per treatment. The soil should be characterized for texture, pH, water-holding capacity, organic matter, and essential nutrients. During cultivation, controlled irrigation with sterile water maintains adequate moisture without causing leaching. Environmental conditions are maintained within species-appropriate ranges: controlled temperature, adjusted photoperiod (generally 12 h light/12 h dark or continuous dark), and moderate ventilation (Jia et al. 2023). Exposure to MPs is continuous, allowing interaction of the particles with the root zone and soil microbiota, enabling assessment of effects on growth, development, and biological functions of the ecosystem. During cultivation, growth and development parameters monitored include plant height, leaf number, leaf area, fresh and dry biomass, root length and branching, and physiological indices such as chlorophyll content, carotenoids, and photosynthetic rate (Sajjad et al. 2022).

In parallel, soil microbiota can be assessed by counting viable microorganisms, molecular analysis of microbial diversity by PCR (Polymerase Chain Reaction), DGGE (Denaturing Gradient Gel Electrophoresis), 16S rRNA sequencing for bacteria and ITS (Internal Transcribed Spacer) for fungi, soil enzymatic activity (dehydrogenase, urease, and phosphatase), and estimation of microbial biomass by fumigation-extraction (Wagg et al. 2014). These analyses allow the correlation of the effects of treatments on the microbiota with plant growth and development, being essential in ecotoxicological studies of microplastics and other emerging contaminants.

## Toward a more accurate environmental assessment

The sustainability assessment of biodegradable materials is often limited to analyzing their decomposition rate in controlled environments. However, this approach overlooks a fundamental aspect: the by-products generated during the degradation process can have significant ecotoxicological effects. Ideally, biodegradation culminates in the complete mineralization of the polymer into carbon dioxide, water, and biomass. However, intermediate steps can release soluble organic compounds, oligomers, additives, or degradation products that have phytotoxic and cytotoxic potential.

Although comprehensive case studies specifically focused on bio-based packaging are still limited due to the relatively recent widespread application of these materials, valuable insights can be drawn from research on the degradation of conventional plastics and the release of additives. In the context of conventional plastics and microplastics, it has already been demonstrated that their decomposition can leach chemical compounds that negatively impact soil health and plant development (Wang et al. 2022; Teng et al. 2022; Li et al. 2024). It is plausible that similar, or even unique, phytotoxic compounds may be generated during the degradation of certain bio-based polymers or their additives, highlighting the need for careful scrutiny beyond merely assessing the rate and extent of decomposition.

Recent studies demonstrate that bioplastics and plant-based materials are not intrinsically safe from a chemical perspective. Although materials such as PLA and PHB are promoted as environmentally friendly, studies show that mere biodegradability does not guarantee the absence of ecotoxicological impacts. In the investigation conducted by Zimmermann et al. (2020), 67% of samples of biodegradable and bio-based materials showed basal toxicity, 42% induced oxidative stress, and 23% showed antiandrogenic activity in in vitro bioassays (Zimmermann et al. 2020). Toxic compounds have been detected in packaging made of PLA, starch, cellulose, and even in materials labeled “safe for food contact.” The study also revealed a massive presence of unidentified chemical characteristics (up to 20,000 per sample), indicating an underestimated chemical complexity in these materials.

In the study conducted by Liwarska-Bizukojc (2022), the biopolymers PLA (polylactic acid) and PHB (polyhydroxybutyrate) did not affect seed germination in test plants, even at high concentrations of up to 11.9% by mass in soil. However, root growth inhibition was frequently observed, especially in dicotyledonous plants such as *Lepidium sativum* (watercress) and *Sinapis alba* (mustard) (Liwarska-Bizukojc 2022). This inhibition was more pronounced with bio-based plastics than with polypropylene (PP), a conventional plastic. Furthermore, it was observed that the effects varied with concentration, polymer type, and plant species, suggesting that released compounds or physicochemical changes in the soil resulting from the presence of plastics influenced plant development. The author herself hypothesizes that negative effects on plant growth may be related to the presence of degradation by-products of these biopolymers or additives in their formulations. Even if complete degradation of PLA and PHB does not occur within the short time span of the tests (72 h), the initial release of soluble compounds or the modification of the soil’s physical properties are sufficient to induce negative effects on root and stem growth, highlighting the importance of considering not only the degradation rate but also the intermediate effects of the process.

Thus, the presence of additives such as dyes, plasticizers, nanoparticles, or antimicrobial agents in biodegradable materials can result in the formation of soluble compounds with adverse effects on plant biota, even when the materials are applied with sustainable intentions. This reinforces the need for more comprehensive environmental assessment protocols, including phytotoxicity testing across different plant species and plant life cycle stages, with special attention to early growth stages.

To illustrate the potential complexities arising from large-scale disposal of biodegradable materials, consider a hypothetical scenario involving the disposal of tons of packaging composed of chitosan, starch, glycerol, silver nanoparticles, and essential oils in a landfill lacking controlled temperature, microbial population management, and proper leachate drainage. Although these materials are generally regarded as non-toxic and biodegradable, the anaerobic conditions prevalent in such a landfill could trigger several adverse effects: (1) inhibited biodegradation: the lack of sufficient oxygen would hinder the activity of aerobic microorganisms, which are primarily responsible for efficient biodegradation; (2) methane generation: anaerobic degradation processes could result in substantial methane production, a potent greenhouse gas, thereby contributing to atmospheric greenhouse gas emissions; (3) leachate formation**:** even if the original materials are non-toxic, incomplete degradation could lead to the formation of organic by-products that may leach into groundwater, posing a risk of water contamination; (4) alteration of soil chemistry**:** the decomposition of such a large volume of organic matter has the potential to alter the pH and chemical composition of the soil surrounding the landfill, potentially impacting adjacent ecosystems.

The chemical variability between batches of the same biopolymer, such as PLA, reveals that the polymer type is not a good predictor of the toxicity of the final product, which is strongly dependent on the formulation, additives, and industrial processes involved. Zimmermann’s research also showed that final products contain more toxic substances than the raw material pellets, indicating that toxicity can be introduced or amplified during industrial processing, including printing, coloring, lamination, and coating application.

**Fig. 3:**
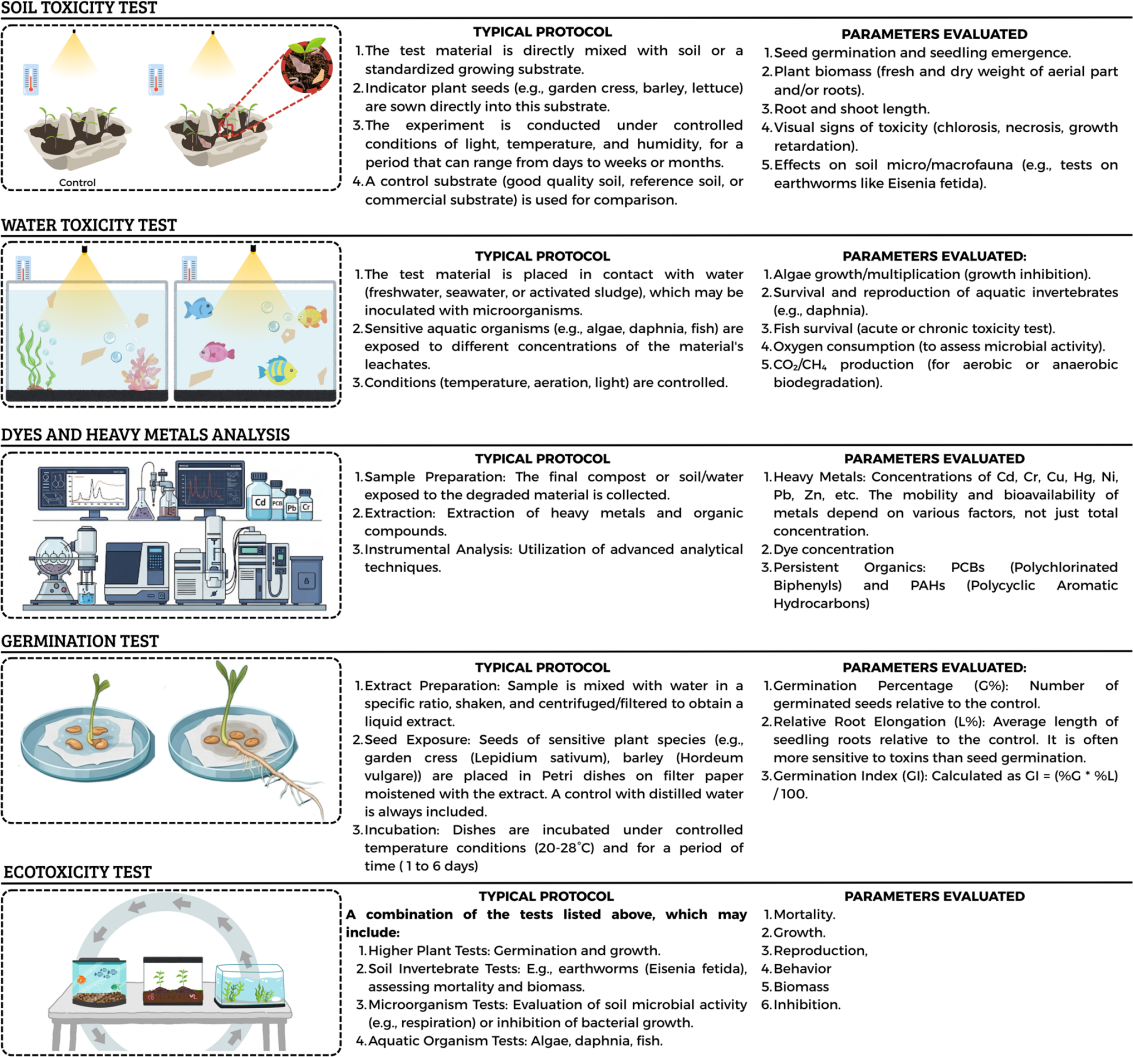
Integrated ecotoxicological and phytotoxicity protocols for assessing biopolymer-based packaging

Therefore, simply labeling a material as “biodegradable” or “plant-based” does not guarantee its environmental benefit. It is essential that environmental assessments include multitrophic ecotoxicity testing, with a special focus on phytotoxicity and cumulative effects on soil, going beyond traditional mass loss or CO₂ evolution tests. Beyond biodegradation and phytotoxicity assays, complementary ecotoxicological approaches, such as soil and aquatic toxicity tests, leachate/contaminant analysis, and integrated ecotoxicity protocols, provide additional evidence of environmental safety for biopolymer-based packaging. Figure 3 summarizes typical protocols and key outcome parameters for these assays, illustrating how a combined strategy yields a more realistic and comprehensive picture of environmental compatibility. These assays not only assess germination rates, root elongation, and biomass accumulation but also provide insights into microbial respiration, invertebrate survival, fish toxicity, and the mobility of heavy metals.

Only with this level of rigor will it be possible to avoid environmentally misleading substitutions and ensure that new materials are aligned with the principles of the circular economy and green chemistry.

## Challenges in assessing the environmental safety of biodegradable packaging

The growing demand for biodegradable materials has driven an urgent need for reliable and comprehensive methodologies to assess their environmental degradation and ecotoxicological impacts. Despite the optimism surrounding the transition to more sustainable packaging, the methods currently employed for such assessments face several significant challenges that must be overcome to ensure the true sustainability of these materials.

One of the main obstacles, and perhaps the most fundamental, is the lack of standardization across testing protocols. Although internationally recognized standards exist, such as those from ASTM (American Society for Testing and Materials) and ISO (International Organization for Standardization), which provide guidelines for assessing biodegradation in specific environments, such as soil, composting, and aquatic systems, the practical implementation and interpretation of these methods still vary widely. Academic research often employs customized methodologies, and commercial certifications may apply distinct criteria regarding experimental conditions, exposure time, temperature, humidity, and the type of soil or compost used. This high degree of variability hinders comparability across studies and the universal interpretation of results, creating confusion in the marketplace and among consumers. The absence of a harmonized regulatory framework that establishes universal validation criteria and specifications for each specific biopolymer across different environments is a critical gap that must be addressed.

Another critical challenge concerns the environmental representativeness of biodegradation tests. Many assessments are conducted under highly controlled and optimized laboratory conditions, such as constant elevated temperatures, ideal humidity levels, and high concentrations of microorganisms, that do not accurately reflect the complexity and variability of natural environments. In soil and aquatic systems, factors such as the diversity and activity of the local microbiota, seasonal fluctuations in temperature and moisture, freeze–thaw cycles, the presence of other contaminants, and soil type (e.g., pH, organic matter content) significantly influence the rate and extent of biodegradation. This can lead to underestimation (in unfavorable environments) or overestimation (under ideal laboratory conditions) of the actual degradation of materials when disposed of in the environment, resulting in sustainability claims that may not hold true in practice. The issue of translating laboratory results to real-world field conditions remains unresolved and is a point of considerable controversy.

Furthermore, the increasing complexity of biodegradable materials presents a significant analytical and assessment challenge. The bioplastics sector is currently undergoing rapid expansion, with the production of an ever-growing variety of bio-based and biodegradable polymers, including polymer blends, composites with organic and inorganic fillers, functional additives (such as plasticizers and dyes), and multilayer structures. Each of these components may exhibit distinct degradation behaviors and complex interactions with the microbial environment. Evaluating the biodegradation of such heterogeneous materials requires integrated analytical approaches capable of fully capturing the environmental fate of each component as well as the material as a whole. Simple measurements of CO₂ evolution or mass loss may be insufficient, making it necessary to perform in-depth analyses of the compounds generated during decomposition, along with visual, morphological, and thermal characterization.

As previously discussed, the assessment of phytotoxicity is a critical aspect that is often overlooked. Most studies focus primarily on quantifying biodegradation, such as CO₂ release or mass loss, while neglecting the potential toxic effects of degradation by-products on plant organisms and soil microbiota. The absence of comprehensive ecotoxicity testing may lead to the adoption of materials that, although physically disappear, leave behind harmful residues or negatively alter soil and water chemistry. Instances in which packaging labeled as biodegradable has exhibited phytotoxic effects, such as the release of salts, organic acids, ammonia, or heavy metals during decomposition, highlight the need for careful scrutiny. The question of whether a phytotoxicity test is equivalent to a compost maturity test, or whether the toxicity arises from substances produced during the composting process versus pollutants present in the raw materials, remains a subject of ongoing debate.

## Future perspectives

The widespread adoption of biodegradable packaging materials will only deliver true environmental benefits if their assessment frameworks evolve beyond current limitations. While internationally standardized protocols ensure a certain degree of reproducibility, they remain insufficient to capture the ecological complexity of real disposal scenarios. Three main advances are urgently required.

First, integration of biodegradability and ecotoxicity endpoints must become standard practice. Protocols should not stop at demonstrating ≥ 90% CO₂ evolution under controlled composting conditions; they must also evaluate whether degradation intermediates or additives exert toxic effects on plants, soil microbiota, aquatic organisms, or higher trophic levels. The growing evidence of phytotoxicity caused by biodegradable microplastics (Serrano-Ruiz et al. 2023; Sun et al. 2023; Han et al. 2024) illustrates the risk of promoting materials as “sustainable” based solely on their capacity to disintegrate.

Second, multi-environment and multitrophic testing is essential. Laboratory assays provide reproducibility but lack representativeness of natural variability in soils, waters, and landfills. Complementing them with in situ or mesocosm experiments will allow better prediction of real-world behavior. Moreover, expanding the spectrum of test organisms, plants, earthworms, microbial communities, and aquatic invertebrates can reveal cumulative and indirect effects invisible to single-endpoint analyses. Eco-certification schemes should be updated accordingly, requiring not just compostability but ecological compatibility.

Third, emerging technologies and interdisciplinary tools offer unprecedented opportunities to refine environmental assessments. Metabolomics, transcriptomics, and microbiome sequencing can detect sublethal and long-term impacts of biopolymers on ecosystems. Machine learning models trained with chemical composition and environmental datasets may predict degradation kinetics and toxic by-products with higher accuracy (Lin and Zhang 2025; Brydon et al. 2025; Chen et al. 2025). Smart sensors for real-time monitoring of CO₂/CH₄ evolution or metabolite release can improve both laboratory and field tests. Importantly, material design should shift toward intrinsically safe biopolymers, in which additives and fillers are selected to minimize phytotoxicity even during incomplete degradation.

Ultimately, a paradigm shift is required: from evaluating whether a packaging “disappears” to ensuring that its degradation contributes positively, or at least neutrally, to ecological cycles. We propose the adoption of a new classification criterion, the effective environmental degradability (EED), summarized in Table 4. This framework recognizes a biopolymer as truly sustainable only when it simultaneously satisfies three conditions: (i) complete microbial mineralization within a reasonable timeframe; (ii) absence of acute or chronic phytotoxic and ecotoxic effects; and (iii) environmental performance validated under realistic disposal scenarios.

**Table 4:** Proposed framework for effective environmental degradability (EED)

Module	Criterion	Endpoint/reference standard	Suggested threshold	Notes
Step 1—Mineralization	Microbial conversion of organic C into CO₂/CH₄	ISO 14855, ASTM D5338 (composting); ASTM D5988 (soil); ISO 14851 (aquatic); ASTM D5511/D5526 (anaerobic)	≥ 90% within 180 days (composting/soil/aquatic); ≥ 70% within 6–12 months (anaerobic)	Normalize using cellulose as reference; report t₉₀
Step 2—Eco-safety	Phytotoxicity and ecotoxicity	ISO 11269–2 (plants); OECD 207 (earthworms); OECD 202/203 (aquatic)	≤ 20% effect compared to control in at least 3 species from distinct trophic levels	Assess residues of metals, additives, and degradation by-products
Step 3—Environmental realism	Realistic disposal scenario	Mesocosm or field assays in soil/water/composting	Qualitative consistency with standardized tests	At least one representative field/mesocosm trial is mandatory

Only under such a framework will biodegradable packaging achieve its promise as a cornerstone of sustainable food systems, avoiding environmentally misleading substitutions and ensuring that the green label reflects genuine ecological safety.

## Conclusions

The transition from petroleum-based plastics to biopolymer-based packaging represents a crucial step toward circular and low-carbon economies. However, this review demonstrates that current biodegradation assessments often overestimate environmental benefits by equating physical disintegration with true ecological safety. The persistence of biodegradable microplastics, the formation of toxic degradation intermediates, and the lack of standardized ecotoxicity testing underscore the need for more rigorous and holistic evaluation frameworks. A truly sustainable packaging material must not only degrade rapidly but also ensure that its degradation products pose no risk to plants, microorganisms, or aquatic organisms. The proposed concept of effective environmental degradability (EED) integrates mineralization efficiency, eco-safety, and environmental realism as complementary pillars for assessing biopolymer fate. Future research should prioritize multidisciplinary approaches that couple omics, metabolomics, and machine learning tools to predict degradation behavior and ecological impacts with higher accuracy. Regulatory frameworks must evolve accordingly to incorporate eco-safety criteria and enforce field validation under realistic disposal conditions. Only through this integrated lens will biodegradable packaging truly contribute to environmental protection, preventing greenwashing and consolidating its role as a cornerstone of sustainable food systems.

## Data Availability

All data generated or analyzed during this study are included in this published article.

## References

[CR1] Abdul‐Baki AA, Anderson JD (1973) Vigor determination in soybean seed by multiple criteria 1. Crop Sci 13:630–633. 10.2135/cropsci1973.0011183X0013x00060013xx

[CR2] AFNOR NF T51–800:2015 Association Française de Normalisation (2015) AFNOR NF T51–800:2015 – Plastiques – Spécifications pour les emballages compostables par compostage domestique. AFNOR

[CR3] AFNOR XP T90–375:2012 Association Française de Normalisation (2012) AFNOR XP T90–375:2012 – Evaluation de la biodégradabilité aérobique ultime des matières plastiques dans l’eau douce – Méthode par mesure du dioxyde de carbone libéré. AFNOR

[CR4] Afshar SV, Boldrin A, Christensen TH, Corami F, Daugaard AE, Rosso B, Hartmann NB (2025) Disintegration of commercial biodegradable plastic products under simulated industrial composting conditions. Sci Rep 15:8569. 10.1038/s41598-025-91647-z 40075087 10.1038/s41598-025-91647-z PMC11904191

[CR5] Ammala A, Bateman S, Dean K, Petinakis E, Sangwan P, Wong S, Yuan Q, Yu L, Patrick C, Leong KH (2011) An overview of degradable and biodegradable polyolefins. Prog Polym Sci 36:1015–1049. 10.1016/j.progpolymsci.2010.12.002

[CR6] ASTM D3826-98 (1998) Standard practice for determining degradation end point in degradable polyethylene and polypropylene using a tensile test. ASTM International, West Conshohocken. www.astm.org

[CR7] ASTM D7475–11 ASTM International (2020) ASTM D7475–11 – Standard test method for determining degradation of plastics in accelerated landfill bioreactor systems. ASTM International

[CR8] ASTM D5338–15 ASTM International (2015) ASTM D5338–15 – Standard test method for determining aerobic biodegradation of plastic materials under controlled composting conditions. ASTM International

[CR9] ASTM D6691–17 ASTM International (2017) ASTM D6691–17 - Standard test method for determining aerobic biodegradation of plastic materials in the marine environment by a defined microbial consortium or natural seawater inoculum. ASTM International

[CR10] ASTM D5511–18 ASTM International (2018) ASTM D5511–18 – Standard test method for determining anaerobic biodegradation of plastic materials under high-solids anaerobic-digestion conditions. ASTM International

[CR11] ASTM D5526–18 ASTM International (2018) ASTM D5526–18 - Standard test method for determining anaerobic biodegradation of plastic materials under accelerated landfill conditions. ASTM International

[CR12] ASTM D5988–18 ASTM International (2018) ASTM D5988–18 – Standard test method for determining aerobic biodegradation of plastic materials in soil. ASTM International

[CR13] ASTM D6868–19 ASTM International (2019) ASTM D6868–19 - Standard specification for labeling of end items that incorporate plastics and polymers as coatings or additives with paper and other substrates designed to be aerobically composted in municipal or industrial facilities. ASTM International

[CR14] ASTM D6400–21 ASTM International (2021) ASTM D6400–21 – Standard specification for labeling of plastics designed to be aerobically composted in municipal or industrial facilities. ASTM International

[CR15] ASTM D6954–24 ASTM International (2024) ASTM D6954–24 – Standard guide for exposing and testing plastics that degrade in the environment by a combination of oxidation and biodegradation. ASTM International

[CR16] Barone AS, Maragoni-Santos C, Farias P, Cortat CMG, Maniglia BC, Ongaratto RS, Ferreira S, Fai AFC (2025) Rethinking single-use plastics: innovations, polices, consumer awareness and market shaping biodegradable solutions in the packaging industry. Trends Food Sci Technol. 10.1016/j.tifs.2025.104906

[CR17] Bauer MG, Henkel F, Gürer U, Lieleg O (2024) Bio-based and degradable food packaging materials: Where are they? Adv Mater Interfaces

[CR18] Bher A, Cho Y, Auras R (2023) Boosting degradation of biodegradable polymers. Macromol Rapid Commun 44:10.1002/marc.202200769 10.1002/marc.202200769 36648129

[CR19] Brydon L, Zhang K, Dobbie G, Taskova K, Wicker JS (2025) Predictive modeling of biodegradation pathways using transformer architectures. J Cheminform 17:21. 10.1186/s13321-025-00969-7 39962584 10.1186/s13321-025-00969-7 PMC11834682

[CR20] CEN/TC 261 (2023) Packaging – European Committee for Standardization Technical Committee 261: Standardization in the field of packaging, including definitions, dimensions, performance requirements and tests (1st ed.). Brussels: European Committee for Standardization (CEN). Available at: https://standards.cen.eu

[CR21] Chen S, Xu G, Chen J, Zhang H, Jiang X, Liu Z, Lin Z, Zhang C, Xu L, Zhang J (2025) Predicting the environmental fate of biodegradable mulch films: a machine learning approach for sustainable agriculture. J Hazard Mater 492:138277. 10.1016/j.jhazmat.2025.138277 40245707 10.1016/j.jhazmat.2025.138277

[CR22] Cheng Z, Ning M, Mao K, Nong X, Li J, Xiao N, Zhang X, Ding Q, Wang H, Liu M (2025) Bamboo powder lignocellulose/polybutylene adipate terephthalate biodegradable bioplastic composite film for food packaging materials. Int J Biol Macromol 316:144781. 10.1016/j.ijbiomac.2025.144781 40446996 10.1016/j.ijbiomac.2025.144781

[CR23] Chinglenthoiba C, Lani MN, Anuar ST, Amesho ATT, Priya KL, Santos JH (2025) Microplastics in food packaging: analytical methods, health risks, and sustainable alternatives. J Hazard Mater Adv 18:100746. 10.1016/j.hazadv.2025.100746

[CR24] Davoudpour Y, Schmidt M, Calabrese F, Richnow HH, Musat N (2023) Correction: High resolution microscopy to evaluate the efficiency of surface sterilization of *Zea mays* seeds. PLoS One 18:e0294203. 10.1371/journal.pone.0294203 37922275 10.1371/journal.pone.0294203 PMC10624265

[CR25] Dissanayake PD, Kim S, Sarkar B, Oleszczuk P, Sang MK, Haque MN, Ahn JH, Bank MS, Ok YS (2022) Effects of microplastics on the terrestrial environment: a critical review. Environ Res 209:112734. 10.1016/j.envres.2022.112734 35065936 10.1016/j.envres.2022.112734

[CR26] Ebrahimi S, Fathi M, Kadivar M (2019) Production and characterization of chitosan-gelatin nanofibers by nozzle-less electrospinning and their application to enhance edible film’s properties. Food Packag Shelf Life 22:100387. 10.1016/j.fpsl.2019.100387

[CR27] Ellis RH (2022) Seed ageing, survival and the improved seed viability equation; forty years on. Seed Sci Technol 50:1–20. 10.15258/sst.2022.50.1.s.01

[CR28] EN 13432:2000 European Committee for Standardization (2000) EN 13432:2000 – Requirements for packaging recoverable through composting and biodegradation – test scheme and evaluation criteria for the final acceptance of packaging. CEN

[CR29] European Commission (2019) COM(2019) 640 final – The European Green Deal. Communication from the Commission to the European Parliament, the European Council, the Council, the European Economic and Social Committee and the Committee of the Regions (1st ed.). Brussels: European Commission. Available at: https://eur-lex.europa.eu/legal-content/EN/TXT/?uri=CELEX:52019DC0640

[CR30] European Commission (2022) COM(2022) 677 final – Proposal for a Regulation of the European Parliament and of the Council on Packaging and Packaging Waste, amending Regulation (EU) 2019/1020 and Directive (EU) 2019/904, and repealing Directive 94/62/EC, 1st ed. European Commission, Brussels

[CR31] Fan H, Hong X, Wang H, Gao F, Su Z, Yao H (2025) Biodegradable microplastics affect tomato (*Solanum lycopersicum L.*) growth by interfering rhizosphere key phylotypes. J Hazard Mater 487:137208. 10.1016/j.jhazmat.2025.137208 39842126 10.1016/j.jhazmat.2025.137208

[CR32] Farooq M, Basra SMA, Ahmad N, Hafeez K (2005) Thermal hardening: a new seed vigor enhancement tool in rice. J Integr Plant Biol 47:187–193. 10.1111/j.1744-7909.2005.00031.x

[CR33] Ferreira DCM, Molina G, Pelissari FM (2020a) Effect of edible coating from cassava starch and Babassu flour (Orbignya phalerata) on Brazilian Cerrado Fruits Quality. Food Bioproc Tech 13:172–179. 10.1007/s11947-019-02366-z/Published

[CR34] Ferreira DCM, Molina G, Pelissari FM (2020b) Biodegradable trays based on cassava starch blended with agroindustrial residues. Compos Part B Eng. 10.1016/j.compositesb.2019.107682

[CR35] Ferreira DCM, dos Santos PN, Santos FH, Molina G, Pelissari FM (2023) Sustainability approaches for agrowaste solution: biodegradable packaging and microbial polysaccharides bio-production. Sci Total Environ. 10.1016/j.scitotenv.2023.163922 10.1016/j.scitotenv.2023.163922 37164094

[CR36] Finch-Savage WE, Bassel GW (2016) Seed vigour and crop establishment: extending performance beyond adaptation. J Exp Bot 67:567–591. 10.1093/jxb/erv490 26585226 10.1093/jxb/erv490

[CR37] García-Depraect O, Lebrero R, Rodriguez-Vega S, Bordel S, Santos-Beneit F, Martínez-Mendoza LJ, Borner RA, Borner T, Moñoz R (2022) Biodegradation of bioplastics under aerobic and anaerobic aqueous conditions: kinetics, carbon fate and particle size effect. Bioresour Technol 344:126265. 10.1016/j.biortech.2021.126265 34737051 10.1016/j.biortech.2021.126265

[CR38] Goel V, Luthra P, Kapur GS, Ramakumar SSV (2021) Biodegradable/bio-plastics: myths and realities. J Polym Environ 29:3079–3104. 10.1007/s10924-021-02099-1

[CR39] Han Y, Teng Y, Wang X, Wen D, Gao P, Yan D, Yang N (2024) Biodegradable PBAT microplastics adversely affect pakchoi (*Brassica chinensis L.*) growth and the rhizosphere ecology: focusing on rhizosphere microbial community composition, element metabolic potential, and root exudates. Sci Total Environ 912:169048. 10.1016/j.scitotenv.2023.169048 38061654 10.1016/j.scitotenv.2023.169048

[CR40] He M, Feng Z, Xu Y, Ding H, Ying C, Cai Y, Zhang H (2023) Macro- and microplastic leachates show a slightly toxic effect on seed germination of cotton. Chemosphere 335:139081. 10.1016/j.chemosphere.2023.139081 37263505 10.1016/j.chemosphere.2023.139081

[CR41] Hernández-García E, Vargas M, González-Martínez C, Chiralt A (2021) Biodegradable antimicrobial films for food packaging: effect of antimicrobials on degradation. Foods 10:1256. 10.3390/foods10061256 34205937 10.3390/foods10061256 PMC8228111

[CR42] Hua M, Jiang C, Zahid HM, Pan Y, Li X, Yao L, gao C, Pan G (2025) High-performance biodegradable polylactide films with nano-reinforced stereo-complex polylactide and layered crystalline structures for eco-friendly packaging applications. Int J Biol Macromol 313:144165. 10.1016/j.ijbiomac.2025.144165 40373910 10.1016/j.ijbiomac.2025.144165

[CR43] ISO 11269–2:2012 International Organization for Standardization (2012) ISO 11269–2:2012 – soil quality – determination of the effects of pollutants on soil flora – Part 2: Effects of chemicals on the emergence and growth of higher plants. ISO

[CR44] ISO 14851:1999 International Organization for Standardization (1999) ISO 14851:1999 – Determination of the ultimate aerobic biodegradability of plastic materials in an aqueous medium – method by measuring the oxygen demand in a closed respirometer. ISO

[CR45] ISO 14852:2018 International Organization for Standardization (2018) ISO 14852:2018 – Determination of the ultimate aerobic biodegradability of plastic materials in an aqueous medium – method by measuring the evolved carbon dioxide. ISO

[CR46] ISO 14855–1:2012 International Organization for Standardization (2012) ISO 14855–1:2012 – Determination of the ultimate aerobic biodegradability of plastic materials under controlled composting conditions – Part 1: General method. ISO

[CR47] ISO 17556:2019 International Organization for Standardization (2019) ISO 17556:2019 – Plastics – Determination of the ultimate aerobic biodegradability in soil by measuring the oxygen demand in a respirometer or the amount of carbon dioxide evolved. ISO

[CR48] ISO 20200:2015 International Organization for Standardization (2015) ISO 20200:2015 – Plastics – Determination of the degree of disintegration of plastic materials under simulated composting conditions in a laboratory-scale test. ISO

[CR49] ISO/TC 122 (2023) Packaging – international organization for standardization technical committee 122: Standardization in the field of packaging with regard to terminology, dimensions, performance and testing (1st ed.). Geneva: International Organization for Standardization (ISO). Available at: https://www.iso.org/committee/5372.html

[CR50] Jia L, Liu L, Zhang Y, Fu W, Liu X, Wang Q, Tanveer M, Huang L (2023) Microplastic stress in plants: effects on plant growth and their remediations. Front Plant Sci. 10.3389/fpls.2023.1226484 10.3389/fpls.2023.1226484 PMC10452891 37636098

[CR51] Jung H, Park S, Park S-A, Kin H, Lee M, Park CH, Jegal J, Shin G, Kim HJ (2025) FDA-hydrolysis activity: a pre-screening tool for optimizing compost selection in standardized plastic biodegradation testing. Waste Manag 204:114907. 10.1016/j.wasman.2025.114907 40435848 10.1016/j.wasman.2025.114907

[CR52] Kaur G, Sharma A, Sharma V (2025) Rapid and non-destructive FTIR-chemometric method for polybag analysis: distinguishing biodegradable from non-biodegradable materials. Next Research 2:100111. 10.1016/j.nexres.2024.100111

[CR53] Lakshmi AGS, Saravanakumar MP (2024) Ageing behavior of starch-based food packaging bioplastics in riparian sediments and sediment-derived dissolved organic matter in the soil environment. J Hazard Mater 480:135778. 10.1016/j.jhazmat.2024.135778 39316919 10.1016/j.jhazmat.2024.135778

[CR54] Lasota J, Błońska E, Kempf M, Kempf P, Tabor S (2024) Impact of various microplastics on the morphological characteristics and nutrition of the young generation of beech (*Fagus sylvatica* L.). Sci Rep 14:19284. 10.1038/s41598-024-70046-w 39164338 10.1038/s41598-024-70046-w PMC11336185

[CR55] Laycock B, Nikolić M, Colwell JM, Gauthier E, Halley P, Bottle S, George G (2017) Lifetime prediction of biodegradable polymers. Prog Polym Sci 71:144–189. 10.1016/j.progpolymsci.2017.02.004

[CR56] Li X, Wang R, Dai W, Luan Y, Li J (2023) Impacts of micro(nano)plastics on terrestrial plants: germination, growth, and litter. Plants 12:3554. 10.3390/plants1220554 37896018 10.3390/plants12203554 PMC10609671

[CR57] Li X, Wang X, Ren C, Palansooriya KN, Wang Z, Chang SX (2024) Microplastic pollution: phytotoxicity, environmental risks, and phytoremediation strategies. Crit Rev Environ Sci Technol 54:486–507. 10.1080/10643389.2023.2252310

[CR58] Lin C, Zhang H (2025) Polymer biodegradation in aquatic environments: a machine learning model informed by meta-analysis of structure-biodegradation relationships. Environ Sci Technol 59:1253–1263. 10.1021/acs.est.4c11282 39772517 10.1021/acs.est.4c11282 PMC11755772

[CR59] Lin Z, Xu D, Zhao Y, Sheng B, wu Z, Wen X, Zhou J, Chen G, Lv J, Wang J, G (2025) Micro/nanoplastics in plantation agricultural products: behavior process, phytotoxicity under biotic and abiotic stresses, and controlling strategies. J Nanobiotechnology 23:231. 10.1186/s12951-025-03314-0 10.1186/s12951-025-03314-0 PMC11927206 40114145

[CR60] Liwarska-Bizukojc E (2022) Phytotoxicity assessment of biodegradable and non-biodegradable plastics using seed germination and early growth tests. Chemosphere. 10.1016/j.chemosphere.2021.133132 10.1016/j.chemosphere.2021.133132 34863727

[CR61] Lozano YM, Caesaria PU, Rillig MC (2022) Microplastics of different shapes increase seed germination synchrony while only films and fibers affect seed germination velocity. Front Environ Sci. 10.3389/fenvs.2022.1017349

[CR62] Lucas N, Bienaime C, Belloy C, Queeneudec M, Silvestre F, Nava-Saucedo J (2008) Polymer biodegradation: mechanisms and estimation techniques – a review. Chemosphere 73:429–442. 10.1016/j.chemosphere.2008.06.064 18723204 10.1016/j.chemosphere.2008.06.064

[CR63] Macan GPF, Munhoz DR, Willems LAJ, Monkley C, Lloyd CEM, Hageman J, geissen V, Land BB, Harkes P (2024) Macro- and microplastics leachates: characterization and impact on seed germination. J Hazard Mater 480:136013. 10.1016/j.jhazmat.2024.136013 39423638 10.1016/j.jhazmat.2024.136013

[CR64] Maguire JD (1962) Speed of germination—aid in selection and evaluation for seedling emergence and vigor 1. Crop Sci 2:176–177. 10.2135/cropsci1962.0011183X000200020033x

[CR65] Meng F, Yang X, Riksen M, Xu M, Geissen V (2021) Response of common bean (*Phaseolus vulgaris L.*) growth to soil contaminated with microplastics. Sci Total Environ 755:142516. 10.1016/j.scitotenv.2020.142516 33045612 10.1016/j.scitotenv.2020.142516

[CR66] Moreno BB, Rodrigues BV, Afonso LR, Jimenez PC, Castro IB (2023) High incidence of false biodegradability claims related to single-use plastic utensils sold in Brazil. Sustain Prod Consum 41:1–8. 10.1016/j.spc.2023.07.024

[CR67] Moudache M, Yalaoui-Guellal D, Boukandoul S, Moulahcene L, Dahi H, Hamri T, Skiba M (2025) Improving the antioxidant activity and stability of food biopackaging enriched by *Moringa oleifera* extract to enhance the shelf life of minced meat. LWT 224:117867. 10.1016/j.lwt.2025.117867

[CR68] Nhu TT, Boone L, Guillard V, Chatellard L, Reis M, Matos M, Dewulf J (2024) Environmental sustainability assessment of biodegradable bio-based poly(3-hydroxybutyrate-co-3-hydroxyvalerate) from agro-residues: production and end-of-life scenarios. J Environ Manage 356:120522. 10.1016/j.jenvman.2024.120522 38493645 10.1016/j.jenvman.2024.120522

[CR69] OECD 208 (2006) Terrestrial plant test: seedling emergence and seedling growth. Organisation for Economic Cooperation and Development (OECD), vol 1. Paris 2006

[CR70] OECD 209 (2010) Activated sludge, respiration inhibition test. Organisation for Economic Cooperation and Development (OECD), vol 1. Paris 2010

[CR71] OECD 216 (2000) Nitrogen transformation test. Organisation for Economic Cooperation and Development (OECD), vol 1. Paris 2000

[CR72] OECD 217 (2000) Carbon transformation test. Organisation for Economic Cooperation and Development (OECD), vol 1. Paris 2000

[CR73] OECD 227 (2006) Terrestrial plant test: vegetative vigour. Organisation for Economic Cooperation and Development (OECD), vol 1. Paris 2006

[CR74] OECD 301 A (1992) Ready biodegradability - DOC die-away test. Organisation for Economic Cooperation and Development (OECD), vol 1. Paris 1992

[CR75] OECD 301 B (1992) Ready biodegradability - CO2 evolution test (modified sturm test). Organisation for Economic Cooperation and Development (OECD), vol 1. Paris 1992

[CR76] OECD 301 C (1992) Ready biodegradability - MITI (I) test. Organisation for Economic Cooperation and Development (OECD), vol 1. Paris 1992

[CR77] OECD 301 D (1992) Ready biodegradability - closed bottle test. Organisation for Economic Cooperation and Development (OECD), vol 1. Paris 1992

[CR78] OECD 301 E (1992) Ready biodegradability - modified OECD screening test. Organisation for Economic Cooperation and Development (OECD), vol 1. Paris 1992

[CR79] OECD 301 F (1992) Ready biodegradability - manometric respirometry test. Organisation for Economic Cooperation and Development (OECD), vol 1. Paris 1992

[CR80] OECD 302 A (1981) Inherent biodegradability - modified SCAS test. Organisation for Economic Cooperation and Development (OECD), vol 1. Paris 1981

[CR81] OECD 302 B (1992) Inherent biodegradability - Zahn-Wellens / EMPA test. Organisation for Economic Cooperation and Development (OECD), vol 1. Paris 1992

[CR82] OECD 302 C (1981) Inherent biodegradability - modified MITI (II) test. Organisation for Economic Cooperation and Development (OECD), vol 1. Paris 1981

[CR83] OECD 306 (1992) Biodegradability in seawater. Organisation for Economic Cooperation and Development (OECD), vol 1. Paris 1992

[CR84] OECD 310 (2014) Ready biodegradability - CO2 in sealed vessels (Headspace test). Organisation for Economic Cooperation and Development (OECD), vol 1. Paris 2014

[CR85] Ortega F, Sobral P, Jios JL, Arce VB, García MA (2022) Starch nanocomposite films: migration studies of nanoparticles to food simulants and bio-disintegration in soil. Polymers (Basel) 14:1636. 10.3390/polym14091636 35566806 10.3390/polym14091636 PMC9099942

[CR86] Pelissari FM, Ferreira DC, Louzada LB, Santos F, Corrêa AC, Moreira FKV, Mattoso LH (2018) Starch-based edible films and coatings: an eco-friendly alternative for food packaging. In: Starches for Food Application: Chemical, Technological and Health Properties. Elsevier, pp 359–420

[CR87] Poli V, Lavagnolo MC, Basaglia M, Bonato T, Zanatta S, Modesti M (2025) Assessment of the biodegradability of polylactic acid (PLA) in freshwater using EN ISO 14851:2019: challenges and outcomes. J Hazard Mater 491:137974. 10.1016/j.jhazmat.2025.137974 40117770 10.1016/j.jhazmat.2025.137974

[CR88] Qi Y, Yang X, Pelaez AM, Lwanga EH, Beriot N, Gertsen H, Garbeva P, Geissen V (2018) Macro- and micro- plastics in soil-plant system: effects of plastic mulch film residues on wheat (*Triticum aestivum*) growth. Sci Total Environ 645:1048–1056. 10.1016/j.scitotenv.2018.07.229 30248830 10.1016/j.scitotenv.2018.07.229

[CR89] Ranal MA, Santana D (2006) How and why to measure the germination process? Rev Bras Bot 29:1–11. 10.1590/S0100-84042006000100002

[CR90] Rashidi L (2022) Standards and Guidelines for Testing Biodegradability of Bioplastic. Biodegradable Polymer-Based Food Packaging. Springer Nature Singapore, Singapore, pp 297–325

[CR91] Sahasa S, Shorobi SS, Mészáros E (2023) Effect of polyethylene microplastics on seed germination of Blackgram (Vigna mungo L.) and Tomato (Solanum lycopersicum L.). Environ Adv11(6):100349. 10.1016/j.envadv.2023.100349

[CR92] Sajjad M, Huang Q, Khan S, Han MA, Liu Y, Wang J, Lian F, Wang Q, Guo G (2022) Microplastics in the soil environment: a critical review. Environ Technol Innov 27:102408. 10.1016/j.eti.2022.102408

[CR93] Samneingjam K, Mahajaroensiri J, Kanathananun M, Aranda CV, Muñoz M, Limwongsaree S (2025) Enhancing polypropylene biodegradability through additive integration for sustainable and reusable laboratory applications. Polymers (Basel) 17:639. 10.3390/polym17050639 40076131 10.3390/polym17050639 PMC11902804

[CR94] Sanna M, Gilardi G, Gullino ML, Mezzalama M (2022) Evaluation of physical and chemical disinfection methods of Brassica oleracea seeds naturally contaminated with Xanthomonas campestris pv. campestris. J Plant Dis Prot 129:1145–1152. 10.1007/s41348-022-00635-2

[CR95] Sarasa J, Gracia JM, Javierre C (2009) Study of the biodisintegration of a bioplastic material waste. Bioresour Technol 100:3764–3768. 10.1016/j.biortech.2008.11.049 19138515 10.1016/j.biortech.2008.11.049

[CR96] Schmid C, Cozzarini L, Zambello E (2021) A critical review on marine litter in the Adriatic Sea: focus on plastic pollution. Environ Pollut 273:116430. 10.1016/j.envpol.2021.116430 33497942 10.1016/j.envpol.2021.116430

[CR97] Seo Y, Chevali V, Lai Y, Zhou Z, Chen G, Burey P, Wang S, Song P (2025) Microplastics in soils: a comparative review on extraction, identification and quantification methods. J Environ Manag 377:124556. 10.1016/j.jenvman.2025.124556 10.1016/j.jenvman.2025.124556 39987865

[CR98] Serrano-Ruiz H, Martin-Closas L, Pelacho AM (2023) Impact of buried debris from agricultural biodegradable plastic mulches on two horticultural crop plants: tomato and lettuce. Sci Total Environ 856:159167. 10.1016/j.scitotenv.2022.159167 36202362 10.1016/j.scitotenv.2022.159167

[CR99] Shi W, Wu N, Zhang Z, Liu Y, Chen J, Li J (2024) A global review on the abundance and threats of microplastics in soils to terrestrial ecosystem and human health. Sci Total Environ 912:169469. 10.1016/j.scitotenv.2023.169469 38154650 10.1016/j.scitotenv.2023.169469

[CR100] Shruti VC, Pérez-Guevara F, Roy PD, Elizalde-Martínez I, Kutralam-Muniasamy G (2020) Identification and characterization of single use oxo/biodegradable plastics from Mexico City, Mexico: is the advertised labeling useful? Sci Total Environ 739:140358. 10.1016/j.scitotenv.2020.140358 32758970 10.1016/j.scitotenv.2020.140358

[CR101] Strotmann U, Durand M, Thouand G, Eberlein C, Heipieper HJ, Gartiser J, Pagga U (2024) Microbiological toxicity tests using standardized ISO/OECD methods—current state and outlook. Appl Microbiol Biotechnol 108:454. 10.1007/s00253-024-13286-0 39215841 10.1007/s00253-024-13286-0 PMC11365844

[CR102] Sun H, Shi Y, Zhao P, Lon G, Li G, Wang J, Qiu D, Lu C, Ding Y, Liu L, He S (2023) Effects of polyethylene and biodegradable microplastics on photosynthesis, antioxidant defense systems, and arsenic accumulation in maize (*Zea mays* L.) seedlings grown in arsenic-contaminated soils. Sci Total Environ 868:161557. 10.1016/j.scitotenv.2023.161557 36640877 10.1016/j.scitotenv.2023.161557

[CR103] Teng L, Zhu Y, Li H, Song X, Shi L (2022) The phytotoxicity of microplastics to the photosynthetic performance ad transcriptome profiling of *Nicotiana tabacum* seedlings. Ecotoxicol Environ Saf 231:113155. 10.1016/j.ecoenv.2021.113155 35007831 10.1016/j.ecoenv.2021.113155

[CR104] Vargas-Estrada L, García-Depraect O, Zimmer J, Muñoz R (2025) Analysis of biological treatment technologies, their present infrastructures and suitability for biodegradable food packaging - a review. J Environ Manage 376:124395. 10.1016/j.jenvman.2025.124395 39933383 10.1016/j.jenvman.2025.124395

[CR105] Wagg C, Bender SF, Widmer F, van der Heijden MGA (2014) Soil biodiversity and soil community composition determine ecosystem multifunctionality. Proc Natl Acad Sci USA 111:5266–5270. 10.1073/pnas.1320054111 24639507 10.1073/pnas.1320054111 PMC3986181

[CR106] Wang W, Yuan W, Xu EG, Li L, Zhang H, Yang Y (2022) Uptake, translocation, and biological impacts of micro(nano)plastics in terrestrial plants: progress and prospects. Environ Res 203:111867. 10.1016/j.envres.2021.111867 34389347 10.1016/j.envres.2021.111867

[CR107] Wang X, Chi Y, Song S (2024) Important soil microbiota’s effects on plants and soils: a comprehensive 30-year systematic literature review. Front Microbiol. 10.3389/fmicb.2024.1347745 10.3389/fmicb.2024.1347745 PMC10999704 38591030

[CR108] Xu B, Liu F, Cryder Z, Huang D, Lu Z, He Y, Wang H, Lu Z, Brookes PC, Tang C, Gan J, Xu J (2020) Microplastics in the soil environment: occurrence, risks, interactions and fate – a review. Crit Rev Environ Sci Technol 50:2175–2222. 10.1080/10643389.2019.1694822

[CR109] Yang Y, Li Z, Yan C, Chadwick D, Jones DL, Liu E, Liu Q, Bai R, He W (2022) Kinetics of microplastic generation from different types of mulch films in agricultural soil. Sci Total Environ 814:152572. 10.1016/j.scitotenv.2021.152572 34954175 10.1016/j.scitotenv.2021.152572

[CR110] Yu L, Chen S, Wang J, Qin L, Sun X, Zhang X, Wang M (2024) Environmental risk thresholds and prediction models of Cd in Chinese agricultural soils. Sci Total Environ 906:167773. 10.1016/j.scitotenv.2023.167773 37839484 10.1016/j.scitotenv.2023.167773

[CR111] Yu Y, Chen Y, Wang Y, Xue S, Liu M, Tang DWS, Yang X, Geissen V (2023) Response of soybean and maize roots and soil enzyme activities to biodegradable microplastics contaminated soil. Ecotoxicol Environ Saf 262:115129. 10.1016/j.ecoenv.2023.115129 37315365 10.1016/j.ecoenv.2023.115129

[CR112] Zantis LJ, Adamczyk S, Velmala SM, Adamczyk B, Vijiver MG, Peijnenburg W, Bosker T (2024) Comparing the impact of microplastics derived from a biodegradable and a conventional plastic mulch on plant performance. Sci Total Environ 935:173265. 10.1016/j.scitotenv.2024.173265 38754499 10.1016/j.scitotenv.2024.173265

[CR113] Zimmermann L, Dombrowski A, Völker C, Wagner M (2020) Are bioplastics and plant-based materials safer than conventional plastics? In vitro toxicity and chemical composition. Environ Int. 10.1016/j.envint.2020.106066 10.1016/j.envint.2020.106066 32951901

